# The Infection Efficiency and Replication Ability of Circularized HBV DNA Optimized the Linear HBV DNA *in Vitro* and *in Vivo*

**DOI:** 10.3390/ijms16035141

**Published:** 2015-03-05

**Authors:** Xiaosong Li, Junke Zhu, Guoqi Lai, Lei Yan, Jieli Hu, Juan Chen, Ni Tang, Ailong Huang

**Affiliations:** 1 Laboratory of Molecular Biology on Infectious Diseases and Institute for Viral Hepatitis, Ministry of Education, Chongqing Medical University, Chongqing 400016, China; E-Mails: xiaosong.lee2015@gmail.com (X.L.); coco.zhu2015@gmail.com (J.Z.); aleex.lee2015@gmail.com (J.H.); wangdan19910601@gmail.com (J.C.); 2 Laboratory Animal Center, Chongqing Medical University, Chongqing 400016, China; E-Mails: a68895078@21cn.com (G.L.); yanlei1028@21cn.com (L.Y.)

**Keywords:** hepatitis B virus (HBV), circularized HBV DNA, covalently closed circular DNA (cccDNA), hepatocellular carcinoma (HCC), persistent infection, viral replication

## Abstract

Studies on molecular mechanisms of the persist infection of hepatitis B virus have been hampered by a lack of a robust animal model. We successfully established a simple, versatile, and reproducible HBV persist infection model *in vitro* and *in vivo* with the circularized HBV DNA. The cells and mice were transfected or injected with circularized HBV DNA and pAAV/HBV1.2, respectively. At the indicated time, the cells, supernatants, serum samples, and liver tissues were collected for virological and serological detection. Both *in vitro* and *in vivo*, the circularized HBV DNA and pAAV/HBV1.2 could replicate and transcribe efficiently, but the infection effect of the former was superior to the latter (*p* < 0.05). The injection of circularized HBV genome DNA into the mice robustly supported HBV infection and approximately 80% of HBV infected mice established persistent infection for at least 10 weeks. This study demonstrated that the infection efficiency and replication ability of the circularized structure of HBV DNA overmatched that of the expression plasmid containing the linear structure of HBV DNA *in vitro* and *in vivo*. Meanwhile, this research results could provide useful tools and methodology for further study of pathogenic mechanisms and potential antiviral treatments of human chronic HBV infection *in vitro* and *in vivo*.

## 1. Introduction

Hepatitis B virus (HBV) is a small (3.2-kb), relaxed circular, partially double-stranded enveloped DNA virus that infects the hepatocytes of humans and other quadrumana [1], which is a major worldwide health problem with more than two billion infected people and 400 million chronic carriers, who are at high risk for developing acute and chronic hepatitis, liver cirrhosis, and hepatocellular carcinoma (HCC), which leads to more than one million deaths annually [2,3,4]. However, due to lack of a small, reproducible, and convenient HBV animal model, the study of the pathogenic mechanisms of HBV infection and the efficiency of antiviraltherapy are still incompletely resolved. The HBV transgenic mice is by far the most commonly used animal model, but this animal model integrates the HBV genome into the mouse chromosome, which inherently increases tolerance to transgenic products [5]. This model does not lead to liver inflammation and forms covalently closed circular DNA (cccDNA) [6]. Scholars have attempted to establish a non-transgenic mice model of HBV, however up until now, these models only caused acute hepatitis [7]. Therefore, scientists are constantly trying to establish animal models of HBV persistent infection. Although the HBV hydrodynamic model has gained unprecedented development, it is difficult to master the plasmid building technology of the hydrodynamic method; thus, it is difficult to establish persistent infection in this model completely [8,9]. The animal model of persistent HBV infection has become the bottleneck of HBV research. The HBV cccDNA mainly exists in hepatocytes; it is the template of HBV mRNA and pgRNA, which plays an important role in the process of HBV replication and relates to HBV persistent infection closely [10]. So far, the study of HBV commonly used *in vitro* and *in vivo* models with linear HBV additions of 1.1 times or 1.2 times the DNA, which was inserted into the eukaryotic expression vector, then transfected or injected to the liver cancer cell line or mice, which could build relatively stable cell lines or animal models to study [11,12,13,14,15,16], however, the infection efficiency and replication ability of the linear HBV DNA *in vitro*, and especially *in vivo*, was relatively insufficient.

It is reported that blood is a much more accessible source of the HBV genome than the liver in hepatitis B patients. The virion-associated HBV DNA is composed of a full-length minus strand and a plus strand of variable length, and it has a relaxed circular configuration. Some scientists have developed a method to amplify the full-length HBV genome using a primer pair targeting the highly conserved precore region, which happens to be present at both the 5' and 3' ends of the minus-strand DNA [17]. Ten years ago, Parekh and colleagues slightly modified the PCR (Polymerase Chain Reaction) primers such that the unique restriction sites incorporated into the sense and antisense primers permit the directional cloning of the PCR product [18].

In this study, the circularized HBV DNA could highly simulate the HBV cccDNA biological behavior in hepatocytes to a certain extent [19], which could better reflect the biological changes of HBV infection in hepatocytes. In this research, we extracted HBV DNA from the patient serum and use the complete primer for PCR amplification and then cyclize to cccDNA. Then we successfully established animal models of persistent HBV infection in immunodeficient and immunocompetent mice by hydrodynamic injection in the mouse tail vein [20]; the hydrodynamic injection of the circularized HBV DNA, which has the self-replicating ability, could lead to HBV persistence for 10 weeks. This animal model expressed HBV DNA, HBcAg, HBeAg and HBsAg, both in hepatocytes and serum, but the serum alanine aminotransferase (ALT) was normal and the inflammation in the liver was slight. The key features of this animal model for HBV persistence are similar to chronic HBV patients [21,22]. Through our research, the circularized HBV DNA could replicate and transcribe efficiently both *in vitro* and *in vivo*. The results could provide useful tools and methodology for further study of pathogenic mechanisms and potential antiviral treatments of human chronic HBV infection.

## 2. Results

### 2.1. Assessment of the Optimal Amount of Circularized HBV DNA and pAAV/HBV1.2 in Vitro and in Vivo

**Figure 1:**
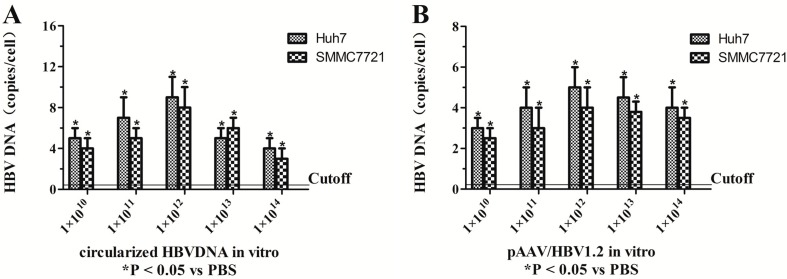

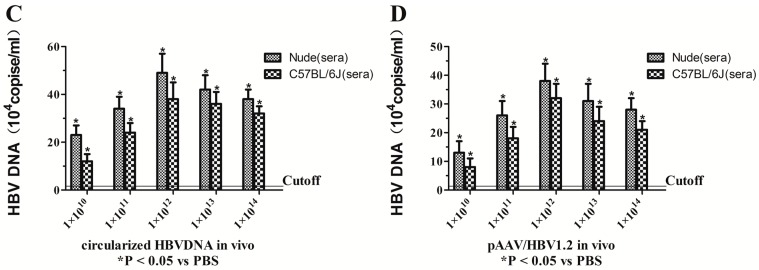
The expression and replication of circularized HBV DNA and pAAV/HBV1.2 were detected *in vitro* and *in vivo* by qPCR (quantitative Polymerase Chain Reaction). (**A**) The expression of circularized HBV DNA *in vitro* (qPCR); (**B**) the expression of pAAV/HBV1.2 *in vitro* (qPCR); (**C**) the expression of circularized HBV DNA *in vivo* (qPCR); and (**D**) the expression of pAAV/HBV1.2 *in vivo* (qPCR). Values present the mean ± standard deviation of three independent experiments. * *p* < 0.05, *vs.* PBS (Phosphate Buffer Solution).

The expression and replication of circularized HBV DNA and pAAV/HBV1.2 has been proved *in vitro* and *in vivo* in early trials. *In vivo*, the HBV1.2 supergenomic DNA could express and replicate in mice hepatocytes [23]. *In vitro*, the concentration gradient of circularized HBV DNA and pAAV/HBV1.2 were 1 × 10^8^, 1 × 10^9^, 1 × 10^10^, 1 × 10^11^, 1 × 10^12^ viral genome/well (six-well plates), respectively. *In vivo*, mice were divided into five groups (five in each group) and injected by various amount of circularized HBV DNA (1 × 10^10^, 1 × 10^11^, 1 × 10^12^, 1 × 10^13^ and 1 × 10^14^ viral genome/2 mL/mouse) and pAAV/HBV1.2 (1 × 10^10^, 1 × 10^11^, 1 × 10^12^, 1 × 10^13^ and 1 × 10^14^ viral genome/2 mL/mouse), respectively. The HBV DNA *in vitro* and *in vivo* (the serum samples were collected via the tail vein after seven days after injection) was detected by real-time fluorescent quantitative PCR. The results of three independent trials showed that the optimal amount of circularized HBV DNA and pAAV/HBV1.2 *in vitro* were both 1 × 10^10^ viral genome/well. The optimal amount of circularized HBV DNA and pAAV/HBV1.2 *in vivo* were 1 × 10^12^ viral genome/2 mL/mouse (Figure 1).

### 2.2. Transfection and Hydrodynamic Injection of Circularized HBV DNA and pAAV/HBV1.2 Could Lead to Viral Gene Replication and Expression in Vitro and in Vivo

**Figure 2:**
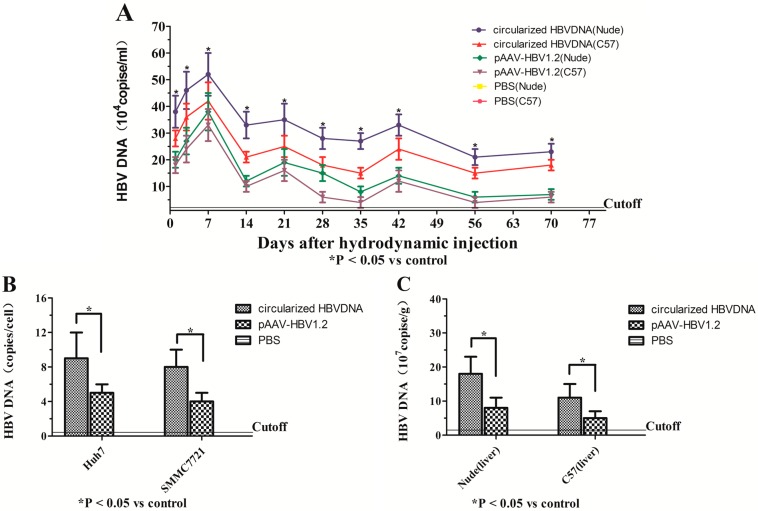

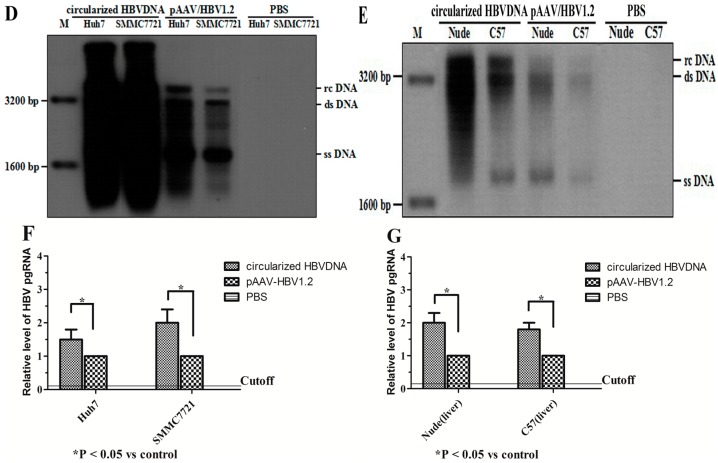
The expression of HBV DNA and HBV pgRNA were detected *in vitro* and *in vivo* by qPCR, Southern blotting (SB) and RT-PCR. (**A**) The levels of HBV DNA in sera (qPCR); (**B**) The levels of HBV DNA in the Huh7 and SMMC7721 cells (qPCR); (**C**) The levels of HBV DNA in hepatic tissue of mice (qPCR); (**D**) The levels of HBV replication intermediates in the Huh7 and SMMC7721 cells (SB); (**E**) The levels of HBV replication intermediates in livers (SB); (**F**) The levels of HBV pgRNA in the Huh7 and SMMC7721 cells; (**G**) The levels of HBV pgRNA in hepatic tissue of mice. Values present the mean ± standard deviation of three independent experiments. * *p* < 0.05, *vs.* control. bp, base pairs; rc DNA, relaxed circularized DNA; ds DNA, double-stranded DNA; ss DNA, single-stranded DNA. RT-PCR, Reverse Transcription-Polymerase Chain Reaction.

The HBV gene expression and replication was tested by real-time fluorescent quantitative PCR and Southern blot (the total DNA was extracted from the cells, sera and livers of mice). As shown by the real-time fluorescent quantitative PCR assay and Southern blot analysis (Figure 2). *In vivo*, the HBV DNA could be detected on day 1 in all mice except the blank group, the sera HBV DNA of all mice except the blank group rose rapidly within the first week and reached the peak, and then began to decline promptly within a certain range. Most of the HBV inside the liver were cleared, especially the C57BL/6J mice; 100% of the injected circularized HBV DNA mice (nude), 80% of the injected circularized HBV DNA mice (C57BL/6J), 60% of the injected pAAV/HBV1.2 mice (nude) and 40% of the injected pAAV/HBV1.2 mice (C57BL/6J) could persist to express the HBV DNA for 10 weeks. The levels of HBV DNA in sera of the injected circularized HBV DNA mice were much higher than the injected pAAV/HBV1.2 mice (*p* < 0.05), which means that the replication level of circularized HBV DNA optimized the pAAV/HBV1.2 in mice, the infection efficiency of HBV in the immunocompetent mice was lower than in the immunodeficient (*p* < 0.05) (Figure 2A). Meanwhile, the study showed that in the transfected Huh7 and SMMC7721 cells, the cyclization of HBV DNA could replicate and transcribe efficiently, and the efficiency and replication ability of the circularized HBV DNA optimized the pAAV/HBV1.2 (Figure 2B). The level of HBV DNA in sera was much lower than in the liver for the same mice (Figure 2C). The HBV replication intermediates (relaxed circularized DNA, double stranded DNA, single stranded DNA) could be detected *in vitro* (Figure 2D) and *in vivo* (Figure 2E), the infection efficiency and replication ability of the circularized structure of HBV DNA overmatched that of the expression plasmid containing the linear structure of HBV DNA *in vitro* and *in vivo*, which was also confirmed by Southern blot analysis (*p* < 0.05). To further detect the levels of HBV RNA, the levels of HBV pgRNA were measured by RT-PCR using primers targeting the 3.5 kb mRNA of the viral genome. The level of HBV pgRNA in the experimental group (circularized HBV DNA) was superior to the positive control group (pAAV/HBV1.2) *in vitro* and *in vivo* (Figure 2F,G).

### 2.3. Expression of HBcAg in Vitro and in Vivo

**Figure 3:**
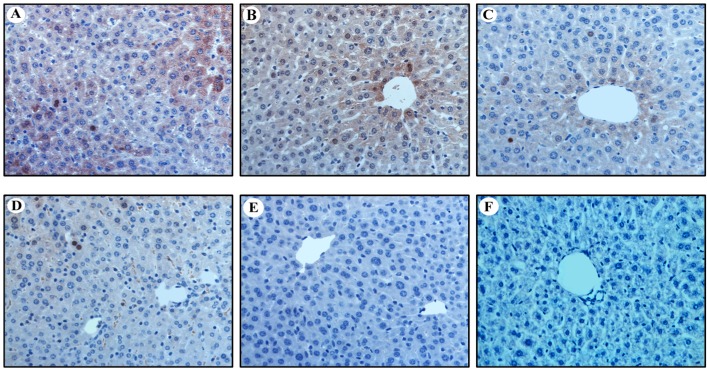
Detection of HBcAg (Hepatitis B Core Antigen) in hepatic tissue of mice at 70 dpi by immunohistochemistry assay (IHC) (Original magnification: ×400). (**A**,**C**,**E**) IHC for HBcAg in liver of nude mice injected with circularized HBV DNA, pAAV/HBV1.2 and PBS, respectively; (**B**,**D**,**F**) IHC for HBcAg in liver of C57BL/6J mice injected with circularized HBV DNA, pAAV/HBV1.2 and PBS, respectively.

**Figure 4:**
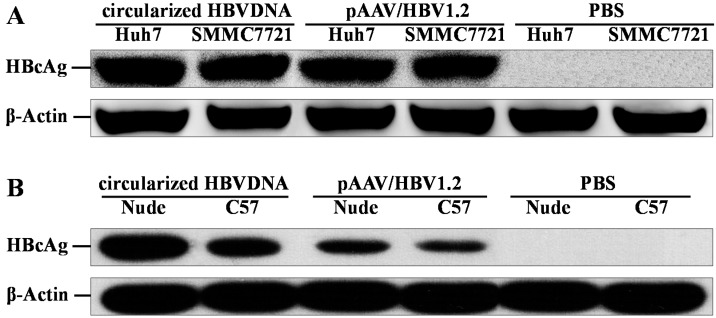
Detection of HBcAg *in vitro* (**A**) and *in vivo* (**B**) by Western blot assay. (**A**) Western blotting results of HBcAg in Huh7 and SMMC7721 cells, which were transfected for five days; and (**B**) Western blotting results of HBcAg in hepatic tissue of mice which were infected for 70 days. β-Actin served as a loading control.

The expression of core protein was detected by IHC and Western blot analysis, as shown in Figure 3 and Figure 4. The HBcAg-positive hepatic cells (both nuclear and cytoplasmic) were randomly interspersed throughout the hepatic lobule with a trend for localization in the central lobule (Figure 3). The HBcAg-positive hepatocytes of nude mice that were injected circularized HBV DNA were the highest (24% ± 8% of hepatocytes were positive; Figure 3A). The HBcAg-positive hepatocytes of C57BL/6J mice that were injected circularized HBV DNA were 13% ± 5% (Figure 3B). The HBcAg-positive hepatocytes of nude mice that were injected pAAV/HBV1.2 was 8% ± 4% (Figure 3C). The HBcAg-positive hepatocytes of C57BL/6J mice that were injected pAAV/HBV1.2 was 5% ± 3% (Figure 3D). The expression of HBcAg in hepatocytes of the injected circularized HBV DNA mice were much higher than the injected pAAV/HBV1.2 mice, which means that the replication level of circularized HBV DNA optimized the pAAV/HBV1.2 in mice (*p* < 0.05). The infection efficiency of HBV in the immunocompetent mice was lower than in the immunodeficient (*p* < 0.05). The HBcAg-positive hepatocytes were surrounded by the inflammatory cells (except the nude mice) and presented partial apoptosis features. The HBcAg could be detected *in vitro* (Figure 4A) and *in vivo* (Figure 4B) by Western blot analysis. The results showed that the levels of HBcAg of the circularized HBV DNA were superior to that of the pAAV/HBV1.2 *in vitro* and *in vivo*, which was also confirmed by Western blot analysis (*p* < 0.05).

### 2.4. Expression of ALT, HBsAg and HBeAg in Vitro and in Vivo

**Figure 5:**
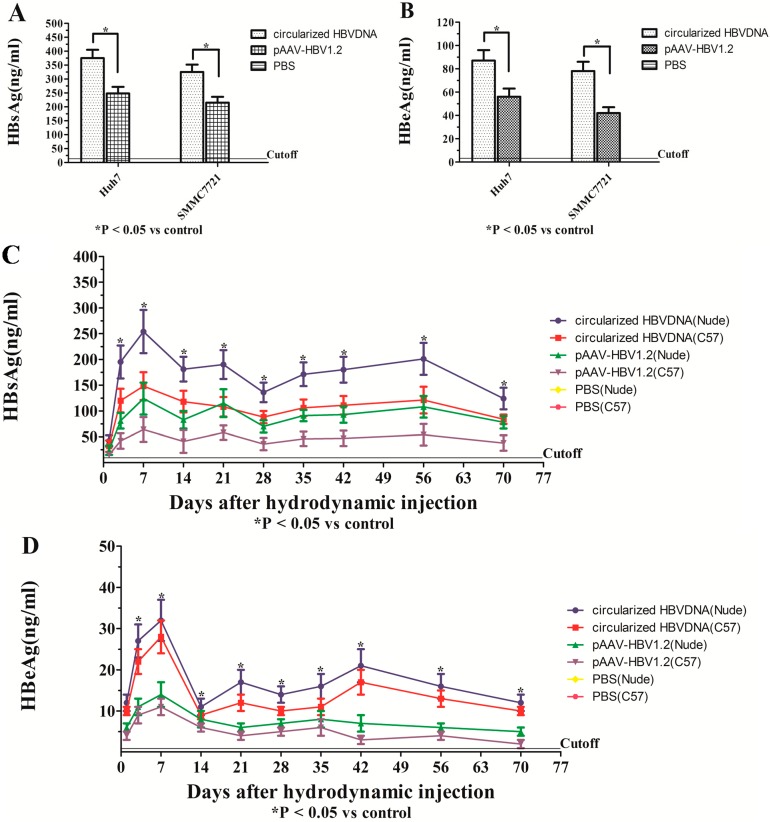
The levels of HBsAg and HBeAg in supernatants and sera were detected by RIA. (**A**) The levels of HBsAg in supernatants at 5 dpi after transfection; (**B**) the levels of HBeAg in supernatants at 5 dpi after transfection; (**C**) the levels of HBsAg in sera after hydrodynamic injection; and (**D**) the levels of HBeAg in sera after injection. HBsAg, Hepatitis B Surface Antigen; HBeAg, Hepatitis B e Antigen; RIA, Radio Immunity Assay; PBS, Phosphate Buffer Solution.

**Figure 6:**
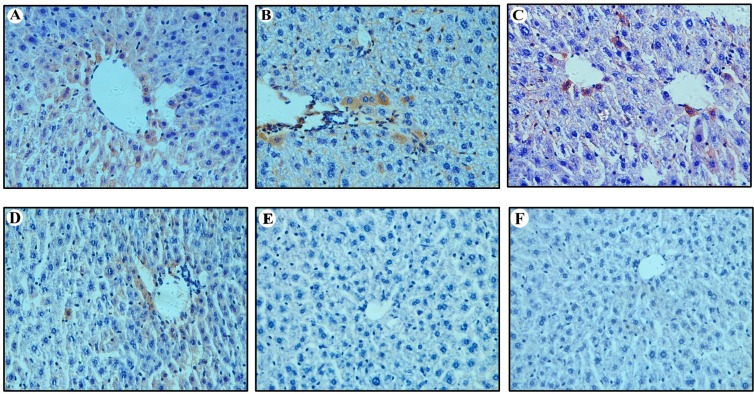
Detection of HBsAg in hepatic tissue of mice at 70 dpi by IHC assay (Original magnification: ×400). (**A**,**C**,**E**) IHC for HBsAg in liver of nude mice injected with circularized HBV DNA, pAAV/HBV1.2 and PBS, respectively; and (**B**,**D**,**F**) IHC for HBsAg in liver of C57BL/6J mice injected with circularized HBV DNA, pAAV/HBV1.2 and PBS, respectively. IHC: Immunohistochemistry.

The level of ALT, HBsAg and HBeAg in serum was detected in a predetermined time by ELISA and RIA, respectively (Figure 5). In this study, the level of the supernants and serum alanine aminotransferase (ALT ranging from 18 to 32 U/L) were not significantly changed (which were not shown). *In vitro*, the HBsAg (Figure 5A) and HBeAg (Figure 5B) could be detected on day 5, except in the PBS group; and the HBsAg and HBeAg expression of the circularized HBV DNA group were higher than the pAAV/HBV1.2 group (*p* < 0.05), both in Huh7 and SMMC7721 cells. *In vivo*, the HBsAg (Figure 5C) and HBeAg (Figure 5D) could be detected on day 1 in all mice, except the blank group; the HBsAg and HBeAg of all mice except the blank group rose rapidly within the first week and reached the peak; and then began to decline promptly within a certain range. The HBsAg and HBeAg expression of nude mice, which were injected circularized HBV DNA was the highest (281.75 and 37.45 ng/mL). In 100% of nude mice that were injected circularized HBV DNA, the HBsAg and HBeAg persisted for 10 weeks. In 80% of C57BL/6J mice that were injected circularized HBV DNA, the HBsAg and HBeAg also persisted for 10 weeks. In 60% of nude mice that were injected pAAV/HBV1.2, the HBsAg and HBeAg persisted for 10 weeks. In 40% of C57BL/6J mice that were injected pAAV/HBV1.2, the HBsAg and HBeAg persisted for 10 weeks. The expression of HBsAg and HBeAg in sera of the injected circularized HBV DNA mice were much higher than the injected pAAV/HBV1.2 mice, which means that the replication level of circularized HBV DNA optimized the pAAV/HBV1.2 in mice (*p* < 0.05), the infection efficiency of HBV in the immunocompetent mice was lower than in the immunodeficient (*p* < 0.05). The expression of HBsAg in hepatic tissue of mice at 70 dpi by IHC showed that the infection efficiency of circularized HBV DNA optimized the pAAV/HBV1.2 *in vivo*. The HBsAg-positive hepatocytes were surrounded by the inflammatory cells (except the nude mice) and presented partial apoptosis features (Figure 6).

### 2.5. Hepatic Histopathological Changes in Mouse Liver

**Figure 7:**
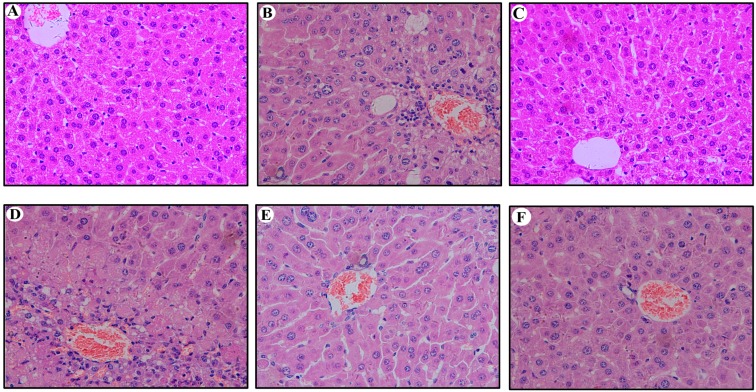
Detection of hepatic histopathological changes in hepatic tissue at 70 dpi by HE assay (Original magnification: ×400). (**A**,**C**,**E**) HE assay for hepatic histopathological changes in liver of nude mice injected with circularized HBV DNA, pAAV/HBV1.2 and PBS, respectively; (**B**,**D**,**F**) HE assay for hepatic histopathological changes in liver of C57BL/6J mice injected with circularized HBV DNA, pAAV/HBV1.2 and PBS, respectively. HE, Hematoxylin-Eosin; PBS, Phosphate Buffer Solution.

**Figure 8:**
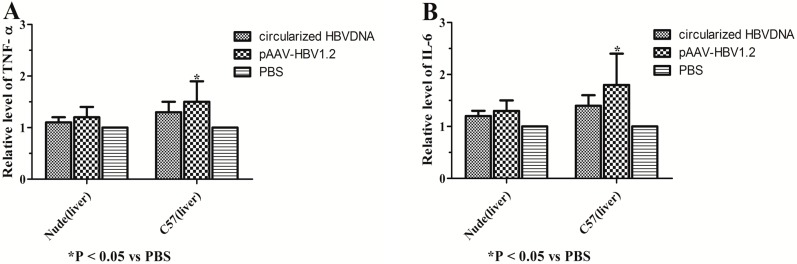
The expression of TNF-α and IL-6 were detected *in vivo* by RT-PCR. (**A**) The levels of TNF-α in the liver; and (**B**) the levels of IL-6 in the liver. Values represent the mean ± standard deviation of three independent experiments. * *p* < 0.05, *vs.* PBS. TNF, Tumor Necrosis Factor; IL, Interleukins.

We further examined whether the HBV clearance in the injected C57BL/6J mice was associated with stronger immune responses compared with those of the nude mice. The livers of C57BL/6J and nude mice at 70 dpi were stained with hematoxylin and eosin. The livers of C57BL/6J mice (except the blank group) at 70 dpi showed multiple foci of mononuclear cell infiltration, but the livers of nude mice (except the blank group) showed slight inflammatory reaction. The HBV plasmid could provoke the obvious immune response to the immunocompetent mice not the immunodeficient mice. Long-term expression of HBV in these carrier mice did not cause severe liver damage, as also evidenced by normal serum alanine aminotransferase levels (ranging from 18 to 32 U/L). The liver tissue of the C57BL/6J and nude mice (except the blank group) exhibited clearly visible lesions. A large number of hepatocytes showed fat degeneration or vacuoles degeneration. The structure of the hepatic lobule and hepatic cord was fuzzy. It was shown that the persistent HBV infection could cause scattered chronic inflammatory cell infiltrates, and the arteries were thickened and surrounded by the inflammatory cells and presented partial apoptosis features (except the blank group) (Figure 7). In addition, we have detected the expression of TNF-α and IL-6 *in vivo* by RT-PCR (Figure 8). The expression of TNF-α and IL-6 in the circularized HBV DNA group and pAAV/HBV1.2 were both higher than the blank group *in vivo*. But there were significant statistically significant differences only in the control group *vs.* the blank group (only in the immunocompetent mice not the immunodeficient mice).

## 3. Discussion

HBV infection is a major public health problem. HBV can be transmitted sexually, percutaneously and perinatally. Approximately 90% of HBV infection in adults can be cleared, whereas 90% of HBV persistent infection in neonates are acquired perinatally [24]. The viral genetic structure and biological function have been studied fully; nevertheless, HBV infection seriously threatens human health as new infections continue to occur. HBV is non-cytopathic, which could lead to cytotoxicity mainly by immune mediation; HBV causes T8 cell subset cytotropism; and HBV intruding into immunological cells is an important reason of cellular immune dysfunction, both in patients and mice [25].

HBV is a hepadnavirus, which is strongly addicted to hepatocytes of humans; the study of HBV pathogenic mechanisms and the new therapies have been hampered by the lack of useful and convenient animal models. Mouse models of HBV infection have significantly aided to our comprehension of the HBV lifecycle and pathogenesis, however the efficiency and stability of HBV infection is very low. HBV transgenic mice were already very mature, but this model will not lead to liver inflammation and formation cccDNA, which is distinct from the process of human infection [26,27].

Due to the high species and tissue specificity of HBV [28], so far, the study of HBV is commonly used in mouse models with linear HBV 1.2 or 1.3 times the DNA, which is inserted into the eukaryotic expression vector, then injected to mice via the tail vein, and acute or chronic HBV infection could be achieved [8,9,29]. However, the efficiency of infection and stability were unsatisfactory. The most important step in HBV DNA replication of hepatocytes is the conversion of the viral rcDNA genome into cccDNA, which is the template for the transcription of viral mRNAs and indicates a successful initiation of infection. Therefore, in this study, the circularized HBV was used to develop stable mouse models of HBV persistent infection. This means that the cyclization of HBV DNA could simulate the actual infection process of HBV, which could lead to acute or chronic HBV infection of humans. In the hepatocytes of humans, the HBV DNA could form cccDNA by covalent ligation of both DNA strands, which was the actual pathological mechanism of chronic HBV infection [19]. There may be 1–50 cccDNA molecules in each infected hepatocyte, which means that the HBV cccDNA is a decisive factor for the persistence of HBV infection; the HBV cccDNA, which only accumulates in the nucleus, serves as a template for the transcription of all the viral mRNAs that indicates a successful initiation of infection [30].

In this experimental system, the PCR product was cloned before transfection, which could enable the unlimited supply of each HBV genome for repeated experiments or further mutagenesis. The circularized HBV DNA (which was circularized by the BspQI-linearized HBV genome with T4 DNA ligase) could greatly improve the replication and expression of core protein. Alternatively, a cryptic polyadenylation signal elsewhere in the HBV genome or in pUC18 vector was used. The transfection of the uncut full-length PCR clones could permit the very efficient expression of viral envelope proteins. As mentioned previously, this increased sensitivity was useful to study immune escape mutants, which harbor amino acid substitutions in the S protein that abolish or diminish its detection by antibodies raised against wild-type protein [31,32,33]. In addition, occult HBV infection also has been attributed to infection by such immune escape mutants [34,35].

Recently, the widely used mouse models with HBV infection were based on hydrodynamic injection of HBV replication-competent plasmids (pAAV/HBV1.2), which produce monomeric linear HBV genome DNA in mouse model [36,37]. In this study, we made some modifications of circularized HBV DNA procedures as mentioned by Qin *et al.* [38] and developed a convenient and efficient animal model for HBV infection, with comparable infection efficiency and replication ability as well as pAAV/HBV1.2. Previous studies have demonstrated that virological properties of HBV isolates were implicated in antiviral response and particular clinical outcomes [39,40]. In comparison to the HBV replication-competent plasmids, circularized HBV DNA were derived from clinical isolates, thus it will be more valuable for evaluating the functional properties of clinical HBV isolates and predicting the response to antiviral drugs. Furthermore, without any foreign sequence and resultant immune response, circularized HBV DNA mimics the natural course of HBV infection and this system provides a valuable tool for the study of HBV virological properties and for selection of antiviral drugs. Taken together, the current HBV mouse model based on circularized HBV DNA helps researchers to determine the impact of host and viral factors on viral replication, expression and pathogenesis, and then assess the antiviral potential of pharmacological agents and physiological processes.

In this study, the HBV persistent infected animal models were built successfully by the hydrodynamic injection of circularized HBV DNA, which was superior to the pAAV/HBV1.2. The specific mechanism may be that the circulating volume in mice has increased dramatically within a very short time-frame, which exceeded the heart load to a great extent. The blood, which included a large number of HBV plasmid accumulated in the hepatic sinusoids, could not backflow, which prolonged the residence time of plasmid DNA in the hepatic sinusoids. Under the conditions of high pressure and large blood loads, the plasmid DNA could be absorbed by the hepatocytes [41]. It could be concluded that the persistent infection of HBV is determined not only by the plasmid backbone but also the mouse genetic background; the infection efficiency of HBV in the immunocompetent mice was lower than in the immunodeficient and the replication level of circularized HBV DNA optimized the plasmids containing linear HBV DNA *in vivo*, the mouse models could help researchers to determine the impact of host and viral factors on HBV pathogenesis, expression and replication, and to assess the antiviral potential of pharmacological agents and physiological processes, including the immune response. There are also some studies that have shown that infection of immune-competent mice with low doses of an adenoviral vector resulted in persistent HBV infection; the mice neither underwent seroconversion production of antibodies against HBV nor developed a strong HBV-specific effector T-cell response [42]. We will explore the specific pathogenic mechanisms and radical treatment for HBV infection. The next plan will be to test different infective doses and routes of HBV DNA, which will be packed in adenovirus, to see if they can stimulate green fluorescent. The plasmid will be microinjected via the hepatic portal vein or tail vein of mice, which may increase the infection efficiency and stability. The real-time dynamic observation of HBV infection could be fulfilled by the green fluorescent in this model. We hope to find the optimal infective dose and route for persistent HBV infection, which could further clarify the pathogenesis of chronic HBV infection and novel antiviral treatment and thoroughly solve this serious threat to human health.

## 4. Experimental Section

### 4.1. Primers

The upstream and downstream of the full-length HBV DNA primers were designed by the Primer 5.0 software; both contained the BspQI endonuclease site [43]. The HBV complete forward primer: 5'-TTATGCTCTTCTTTTTCACCTCTGCCTARTCATC-3', the HBV complete reverse primer: 5'-TCATGCTCTTCAAAAAGTTGCATGGTGCTGGTG-3'. The primers for detection and amplification of the HBV DNA fragments were designed specifically for the conserved region of the HBV gene by the Primer 5.0 software and the sequences were as follows: forward primer (F402): 5'-CCTCTTCATCCTGCTGCT-3'; and reverse primer (R718): 5'-AACTGAAAGCCAAACAGTG-3'. All the primers were synthesized by Invitrogen Bio-Tech Co., Ltd. (Shanghai, China).

### 4.2. Cells

The Huh7 and SMMC7721 cells were provided by the Laboratory of Molecular Biology on Infectious Diseases, Ministry of Education, Chongqing Medical University, Chongqing, China. All the cells were kept as monolayer cultures with Dulbecco’s Modified Eagle’s Medium containing 10% fetal bovine serum, 100 U/mL streptomycin and 100 mg/mL penicillin, which were all provided by the same company (DMEM; Hyclone, Shanghai, China). All the cells were cultured in a humidified incubator with 5% CO_2_ at 37 °C.

### 4.3. Animals

Male nude and C57BL/6J mice (6–8 weeks old, 18–24 g, SPF level) were provided by the Laboratory Animal Center of Chongqing Medical University. The Chongqing Medical University Medical Research of Ethics Committee approved all animal experiments (The Ethics Committee of Chongqing Medical University: 2014015, 5 March 2014). All animals ate rat chow and drank sterile water *ad libitum*, and were kept at a 12 h light–dark cycle at a constant humidity and temperature.

### 4.4. The Preparation of Circularized HBV DNA and pAAV/HBV1.2

**Figure 9:**
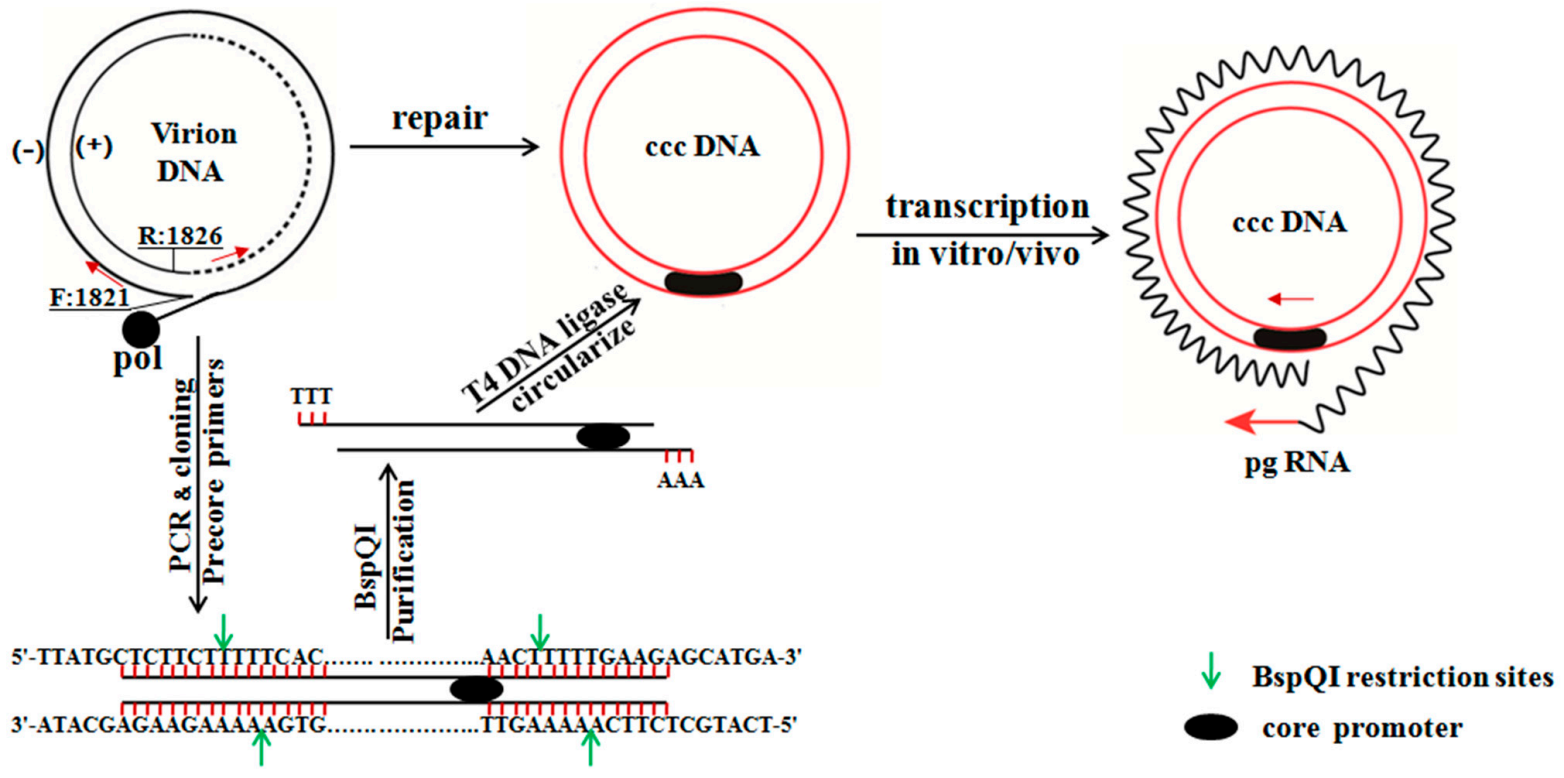
Generation of replication-competent HBV forms (circularized HBV DNA) from full-length PCR product. Primers targeting the precore region permit the amplification of the full-length HBV genome from virion-associated DNA. The replication of the HBV genome requires the transcription of the 3.5-kb terminally redundant pg RNA under the core promoter (shown in a black oval), which is feasible from the cccDNA template, but not from a single copy of the HBV genome cloned to a vector. One approach is to release the HBV insert by digestion with BspQI, followed by the construction of an EcoRI dimer through an intermediate of the precore dimer. A much simpler approach is to use the BspQI digest, which is converted to a circular form by a cellular ligase or, more efficiently, by T4 DNA ligase *in vitro* and *in vivo*.

To establish circularized HBV DNA, the primers for fragment and full-length amplification containing BspQI restrictive enzyme cutting site were designed and HBV DNA from patients (subtype adw) was used as a template. The full-length HBV was acquired by nested PCR combined with sectional amplification. The full-length HBV DNA was cloned into pMD19-T vector. The circularized HBV DNA could be obtained by the restrictive enzyme digestion and T4 DNA ligase ligation. The establishment of pAAV/HBV1.2 was that the HBV1.2 full-length DNA was subcloned from the plasmid pHBV-48, which contains 1.2 copies of the HBV genome (subtype adw) to a rAAV vector, pAAV-GFP [44]. Two fragments (which were digested by EcoRI/BglII and BamHI/EcoRI, respectively) of pHBV-48 were cloned into a BglII site of the AAV-GFP vector. The consequent pAAV/HBV1.2 incorporates the HBV fragment crossing nucleotides 1400~3182/1~1987, which were flanked by the inverted terminal repeats of the AAV. The HBV gene expression cassette, which was located inside the two inverted terminal repeats, was resected by the SmaI digestion of the pAAV/HBV1.2 [16] (Figure 9). The pAAV/HBV1.2 was kindly provided by Prof. Chen PJ (Graduate Institute of Clinical Medicine, College of Medicine, National Taiwan University).

### 4.5. The Transfection of Circularized HBV DNA and pAAV/HBV1.2 to Cells

The Huh7 (1 × 10^5^ cells/well) and SMMC7721 (1 × 10^5^ cells/well) cells were seeded in the six-well plates and cultured overnight at 37 °C with 5% CO_2_. On the following day, the circularized HBV DNA (1 × 10^10^ viral genome/well) and pAAV/HBV1.2 (1 × 10^10^ viral genome/well) were added to the culture medium (the blank group added PBS). When the cell density reached 70%, it was followed by 3 days incubation in the same culture medium and were placed in a humidified incubator with 5% CO_2_ at 37 °C. On the fifth day, the supernatants and cells were collected and counted.

### 4.6. The Hydrodynamic Injection of Circularized HBV DNA and pAAV/HBV1.2 to Mice

Sixty nude mice and sixty C57BL/6J mice were randomly divided into 3 groups (20 mice in each group). The experimental group was injected with circularized HBV DNA (1 × 10^12^ viral genome/2 mL/mouse), the control group was injected with pAAV/HBV1.2 (1 × 10^12^ viral genome/2 mL/mouse), and the blank group was injected with PBS (2 mL). Mice were injected with circularized HBV DNA and pAAV/HBV1.2 (dissolved in 2 mL PBS) via the tail vein within 5–8 s [20]. The injection rate was 0.3 mL/s [45]. The serum samples were collected via the tail vein 1 day, 3 days, 1 week, 2 weeks, 3 weeks, 4 weeks, 5 weeks, 6 weeks, 8 weeks and 10 weeks after injection. The mice were sacrificed after 10 weeks and then the serum and liver tissue were collected.

### 4.7. Detection and Quantification of HBV DNA

In this study, the cells, serum and hepatictissue HBV DNA were detected by real-time fluorescent quantitative PCR. The serum (50 μL) and hepatictissue (20 mg) total DNA was extracted by the TIANamp Virus DNA/RNA Kit (Tiangen Bio-Tech Co., Ltd., Beijing, China) and Wizard^®^ Genomic DNA Purification Kit (Promega, Madison, WI, USA), 2 μg total DNA was used for qPCR. The quantification of HBV copies was performed by SYBR-Green assays using FastStart Universal SYBR-Green Master mix (Roche Diagnostics GmbH, Mannheim, Germany). Primers for amplification of the HBV DNA fragments were designed specifically for the conserved region of the HBV gene by Invitrogen Bio-Tech Co., Ltd. (Shanghai, China) and the sequences were as follows: Forward primer (F402), 5'-CCTCTTCATCCTGCTGCT-3'; and reverse primer (R718), 5'-AACTGAAAGCCAAACAGTG-3'. The standard curve was established by the serum HBV DNA of a hepatitis B patient (as a known titer: 2 × 10^3^, 2 × 10^4^, 2 × 10^5^, 2 × 10^6^, 2 × 10^7^ and 2 × 10^8^ copies/μL). PCR reaction was programmed as follows: 3 min at 95 °C; 40 cycles of 20 s at 94 °C; 30 s at 50 °C (qPCR/Melt data acquisition); and 32 s at 72 °C.

### 4.8. Detection and Quantification of HBV pgRNA

The total RNA of the cells and hepatic tissue were detected by the DNA-free RNA Mini Extraction kit (Watson, Shanghai, China) and 1 μg total RNA was used for cDNA synthesis, which was conducted by reverse transcription using the PrimeScript RT reagent kit (Perfect Real Time; Takara Bio, Inc., Shiga, Japan). The relative quantification of HBV pgRNA was performed by SYBR Green assays, the β-actin mRNA was treated as an endogenous control. The expression values of HBV pgRNA were calculated using the 2^−ΔΔ*C*t^ method.

### 4.9. Detection and Quantification of ALT, HBsAg and HBeAg

The supernatant of the culture medium and serum ALT were detected by the Mouse Alanine Aminotransferase ELISA kit (Bogoo Bio-Tech Co., Ltd., Shanghai, China). The supernatant of the culture medium and serum HBsAg and HBeAg were detected by Diagnostic Kit for Hepatitis B s Antigen and Hepatitis B e Antigen (Radioimmunoassay, Beijing North Biotechnology Research Institute, Beijing, China).

### 4.10. Detection and Quantification of TNF-α and IL-6 in Vivo

The total RNA of the hepatic tissue were detected by the DNA-free RNA Mini Extraction kit (Watson, Shanghai, China) and 1 μg total RNA was used for cDNA synthesis, which was conducted by reverse transcription using the PrimeScript RT reagent kit (Perfect Real Time; Takara Bio, Inc.). Primers for amplification of the TNF-α and IL-6 were synthesized by Invitrogen Bio-Tech Co., Ltd. (Shanghai, China) and the sequences were as follows: forward primer, TNF-α: 5'-CAGCCGATGGGTTGTACCTT-3', IL-6: 5'-GAGAAAAGAGTTGTGCAATGGC-3'; and reverse primer, TNF-α: 5'-GGCAGCCTTGTCCCTTGA-3', IL-6: 5'-ACTAGGTTTGCCGAGTAGACC-3'. The relative quantification of TNF-α and IL-6 was performed by SYBR Green assays, the β-actin mRNA was treated as an endogenous control. The expression values of TNF-α and IL-6 were calculated using the 2^−ΔΔ*C*t^ method.

### 4.11. Hepatic Histopathological and Immunohistochemical Analysis (IHC)

The hepatic histopathological change could be observed by HE (Htoxylin Eosin) staining. The location and expression of virus antigen in liver tissue could be detected by immunohistochemical staining [46]. Paraformaldehyde-fixed paraffin-embedded tissue sections (4.5 μm thickness) were stained with HE and IHC staining. HBsAg and HBcAg were determined in the liver sections by the IHC Staining with mouse anti-HBsAg (ab20402, Abcam, Cambridge, UK) and rabbit anti-HBcAg (B0586, DAKO, Glostrup, Denmark). The negative staining contrasts were stained with PBS only.

### 4.12. Southern Blot Analysis

The HBV replicative intermediates were extracted from the Huh7 and SMMC7721 cells [41]. Meanwhile, the HBV total DNA has been extracted from the hepatocyte by Wizard^®^ Genomic DNA Purification Kit (Promega) and then the DNA were separated by 0.8% agarose gels. The DNA samples were shifted to nitrocellulose membranes (Roche Diagnostics GmbH). The membranes were ultraviolet crosslinked and prehybridized and then the hybridizations were marked by a digoxigenin-labeled HBV-specific probe, which was a Random-Primed DNA Labeling kit (Roche Diagnostics GmbH). The signal was probed by the exposure of an X-ray film and scanned by the Versa Doc Imaging system (Bio-Rad, Hercules, CA, USA).

### 4.13. Western Blot Analysis

The cellular proteins in the Huh7 and SMMC7721 cells were extracted using radio immunoprecipitation buffer supplemented with phenylmethanesulfonyl fluoride. Meanwhile, the fresh liver tissue were fully ground by the tissuelyser-24 (Shanghai Jingxing Science and Technology Co., Ltd., Shanghai, China) and then 1% RIPA Lysis Buffer (Beyotime Institute of Biotechnology, Shanghai, China) was added until the cells were lysed sufficiently. The protein concentration was tested by a bicinchoninic acid assay protein concentration determination kit (Beyotime Institute of Biotechnology, Shanghai, China). Equal quantities of samples were separated by 10% SDS-PAGE and then transferred to a polyvinyl difluoride (PVDF) membrane. The membranes were incubated by polyclonal monoclonal rabbit anti-HBcAg (B0586, DAKO, Denmark). The membranes were washed three times in TBST and then incubated by a goat anti-rabbit secondary antibody (Santa Cruz Biotechnology, Inc., Santa Cruz, CA, USA). The signals were determined by the Enhanced Chemiluminescence Detection system (Pierce Biotechnology, Inc., Rockford, IL, USA) and the β-actin was used to normalize the laboratory data [47].

### 4.14. Statistical Analysis

The data were expressed as mean ± standard deviation (at least three independent experiments). Statistical analysis was performed using the independent *t*-test. The one-way analysis of variance was used to assess the differences between groups and the laboratory data were considered to be statistically significant when *p* < 0.05. All analyses were calculated by the Statistical package of the Social sciences statistical software for windows, version 19.0.1 (SPSS, Inc., Chicago, IL, USA).

## 5. Conclusions

In summary, we successfully evaluated the infection efficiency and replication ability between the circularized and the linear HBV DNA *in vitro* and *in vivo* for the first time. The infection efficiency of HBV DNA in the immunocompetent mice was lower than in the immunodeficient. The infection efficiency and replication ability of the circularized structure of HBV DNA overmatched that of the expression plasmid containing the linear structure of HBV DNA *in vitro* and *in vivo*. The results could provide useful tools and methodology for further study of pathogenic mechanisms and potential antiviral treatments of human chronic HBV infection.
